# Integrative epigenomic analysis reveals unique epigenetic signatures involved in unipotency of mouse female germline stem cells

**DOI:** 10.1186/s13059-016-1023-z

**Published:** 2016-07-27

**Authors:** Xiao-Li Zhang, Jun Wu, Jian Wang, Tingting Shen, Hua Li, Jun Lu, Yunzhao Gu, Yani Kang, Chee-Hong Wong, Chew Yee Ngan, Zhifeng Shao, Ji Wu, Xiaodong Zhao

**Affiliations:** 1 Shanghai Center for Systems Biomedicine, Bio-ID Center, School of Biomedical Engineering, Shanghai Jiao Tong University, Shanghai, 200240 China; 2 Bio-X Institutes, Shanghai Jiao Tong University, Shanghai, 200240 China; 3 Sequencing Technology Group, Joint Genome Institute, Lawrence Berkeley National Laboratory, Walnut Creek, CA 94598 USA; 4 Key Laboratory of Fertility Preservation and Maintenance of Ministry of Education, Ningxia Medical University, Yinchuan, 750004 China

**Keywords:** Female germline stem cell, Epigenome, ChIP-Seq

## Abstract

**Background:**

Germline stem cells play an essential role in establishing the fertility of an organism. Although extensively characterized, the regulatory mechanisms that govern the fundamental properties of mammalian female germline stem cells remain poorly understood.

**Results:**

We generate genome-wide profiles of the histone modifications H3K4me1, H3K27ac, H3K4me3, and H3K27me3, DNA methylation, and RNA polymerase II occupancy and perform transcriptome analysis in mouse female germline stem cells. Comparison of enhancer regions between embryonic stem cells and female germline stem cells identifies the lineage-specific enhancers involved in germline stem cell features. Additionally, our results indicate that DNA methylation primarily contributes to female germline stem cell unipotency by suppressing the somatic program and is potentially involved in maintenance of sexual identity when compared with male germline stem cells. Moreover, we demonstrate down-regulation of Prmt5 triggers differentiation and thus uncover a role for Prmt5 in maintaining the undifferentiated status of female germline stem cells.

**Conclusions:**

The genome-wide epigenetic signatures and the transcription regulators identified here provide an invaluable resource for understanding the fundamental features of mouse female germline stem cells.

**Electronic supplementary material:**

The online version of this article (doi:10.1186/s13059-016-1023-z) contains supplementary material, which is available to authorized users.

## Background

Germline stem cells (GSCs) are of essential importance for genome transmission from generation to generation [1]. Although unipotent, GSCs have a unique capability to continuously generate gametes. In recent years, extensive efforts have been made to understand the specification of primordial germ cells (PGCs) and the profound epigenetic reprogramming (including genome-wide DNA demethylation and histone remodeling) which is necessary for the zygote to acquire totipotency after fertilization [2, 3]. Much less is known about the regulatory mechanisms that govern the fundamental properties of mammalian female GSCs. It has long been believed that female mammals lose the ability to produce oocytes at birth [4–8]. However, this concept has been reshaped by recent studies in which female germline stem cells (FGSCs) have been identified in postnatal ovaries of various mammalian organisms [9–13]. Therefore, more attention needs to be paid to characterizing the mechanisms for the maintenance of unipotency and undifferentiated status of FGSCs, which is critical for understanding GSC biology.

Knowledge on the close coordination of genetic and epigenetic regulation is important for understanding the basic properties of stem cells and their differentiation [2]. In addition to genetic programs directed by lineage-specific transcription factors, genome-wide epigenetic modifications are also actively involved in development and cell fate determination, which constitute another layer of regulation beyond the genome sequence. Epigenetic profiling has been well documented in studies of embryonic stem cells (ESCs) [14]. For example, some promoter regions in ESCs are co-marked by H3K4me3 and H3K27me3 and have been termed bivalent domains. These bivalent genes are poised at the ESC stage and could be activated in downstream development stages [15]. Subsequently, our genome-wide profiling analysis of H3K4me3 and H3K27me3 revealed that the different combinations of histone modifications modulate diverse transcriptional patterns and are involved in the fundamental properties of ESCs [16]. A study of H3K4me1 and H3K27ac, histone modifications marking enhancers, indicated that they are cell type-specific and involved in determining cellular identity [17]. Identifying and characterizing regulatory DNA elements (e.g., promoters and enhancers) is hugely difficult due to the lack of recognizable and consistent sequence features but epigenetic profiling in ESCs has proven that it is a powerful tool to delineate these. Meanwhile, these profiling analyses provide insights into the understanding of stem cell biology.

In previous reports, we generated mouse FGSC lines and demonstrated that FGSCs could undergo oogenesis once transplanted into ovaries of infertile mice and give rise to offspring [9, 13]. Although this study was viewed to be useful for both basic research and medicine [18], the regulatory mechanisms that govern the identity of FGSCs remain elusive. Here, we carried out extensive epigenomic profiling and RNA sequencing (RNA-Seq) analyses with the aim of understanding epigenetic and genetic control in mouse FGSCs.

## Results

### FGSCs exhibit lineage-specific gene expression signatures

We first characterized the cultured FGSCs by examining the molecular signatures associated with germline development. Immunocytochemical analysis indicated that FGSCs are positive for germline-specific markers Mvh and Fragilis (Additional file 1: Figure S1a). Then we examined the expression of other germ cell-specific markers by reverse transcription polymerase chain reaction (RT-PCR). We found that *Dazl* and *Stella* are expressed in FGSCs (Additional file 1: Figure S1b). The characteristics detected in this study are consistent with the observations we reported previously [9, 13].

**Fig. 1:**
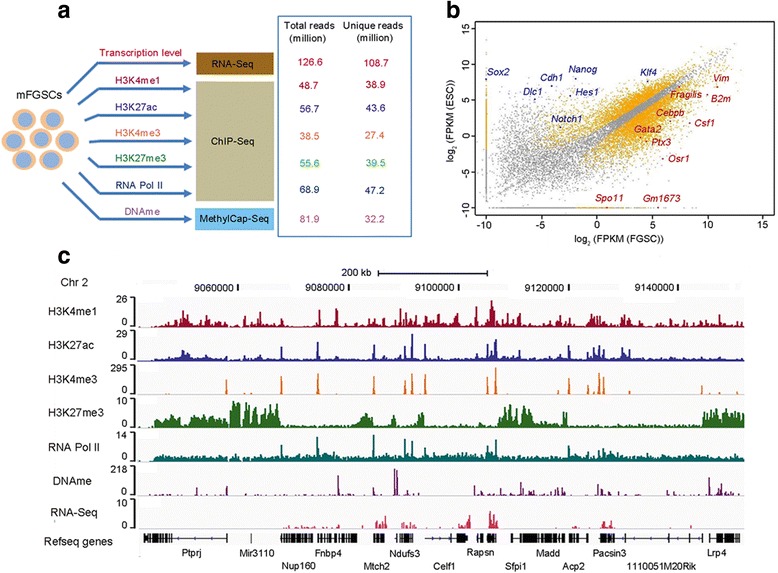
Generation of genome-wide epigenome reference maps in FGSCs. **a** Design and overview of the experimental approaches used for the integrative analyses of the epigenome and transcriptome in mouse FGSCs (*mFGSCs*). **b** Scatter plot of FGSC/ESC expression data sets. *Orange dots* indicate genes with significantly differential expression (*p* < 0.01). **c** A snapshot of the IGV genome browser showing sequencing reads of four histone modifications, DNA methylation, RNA polymerse II (*RNA Pol II*) occupancy, and RNA-Seq in FGSCs

To obtain a global view of the transcription pattern of FGSCs, we performed transcriptional profiling of mRNA with strand-specific RNA-Seq (Fig. 1a) and compared our data with those from ESCs [19]. In contrast to *Nanog* and *Sox2*, which are specifically expressed in ESCs, we found that *Ifitm3*/*Fragilis*, *Ptx3*, and *GM1673* are selectively expressed in FGSCs (Fig. 1b). Moreover, we found that *Akt1* is highly expressed in FGSCs; the Akt1 pathway is involved in self-renewal of mouse germline stem cells [20]. Piwi proteins bind piwi-interacting RNA (piRNA), which is responsible for repetitive element silencing during germline development. Intriguingly, we observed that the piwi proteins Mili, Miwi, and Miwi2 are not actively expressed, probably due to the Piwi–piRNA pathway, which is particularly involved in gametogenesis [21]. Thus, these observations verify the known molecular signatures of FGSCs.

### Extensive mapping of chromatin marks in FGSCs

To explore the chromatin state and its effect on the properties of FGSCs, we performed chromatin immunoprecipitation sequencing (ChIP-Seq) to generate genome-wide maps by profiling four histone modifications (H3K4me1, H3K27ac, H3K4me3, and H3K27me3) and RNA polymerase II (RNA Pol II) occupancy. We also profiled global DNA methylation by MethylCap-Seq. In addition, we measured gene expression levels with RNA-Seq and generated more than 108 million uniquely mapped reads for detecting gene expression in FGSCs (Fig. 1a; Additional file 1: Table S1). For each high-throughput sequencing analysis, at least two biological replicates were performed, which are fairly correlated (Additional file 1: Figure S2a). All data sets have been deposited in a public database.

The sequencing data were visualized in the Integrative Genomics Viewer (IGV) by generating histograms of normalized densities of ChIP fragments across the FGSC genome (Fig. 1c; Additional file 1: Figure S3) [22]. The map of histone modifications and DNA methylation shows signal distributions that are consistent with their functions [14]. For example, H3K4me3 has been regarded as a hallmark of transcription initiation and is primarily localized at promoters [23]. In our study we found 90 % of H3K4me3 sites are located at promoter regions. A case in point is the presence of strong H3K4me3 at the promoter of *Ifitm3*/*Fragilis*, which encodes a protein used to generate the FGSC line [9, 13]. H3K27ac and RNA Pol II are associated with active transcription and we found they are highly enriched at promoter regions in FGSCs (Additional file 1: Figure S2b). Additionally, we observed that H3K27me3 is broadly distributed across the FGSC genome, similar to what we found in ESCs [16]. Taken together, these observations suggest the data sets we generated here are able to be used to identify the *cis*-regulatory elements in FGSCs.

### Active enhancers distinguish FGSCs from ESCs

**Fig. 2:**
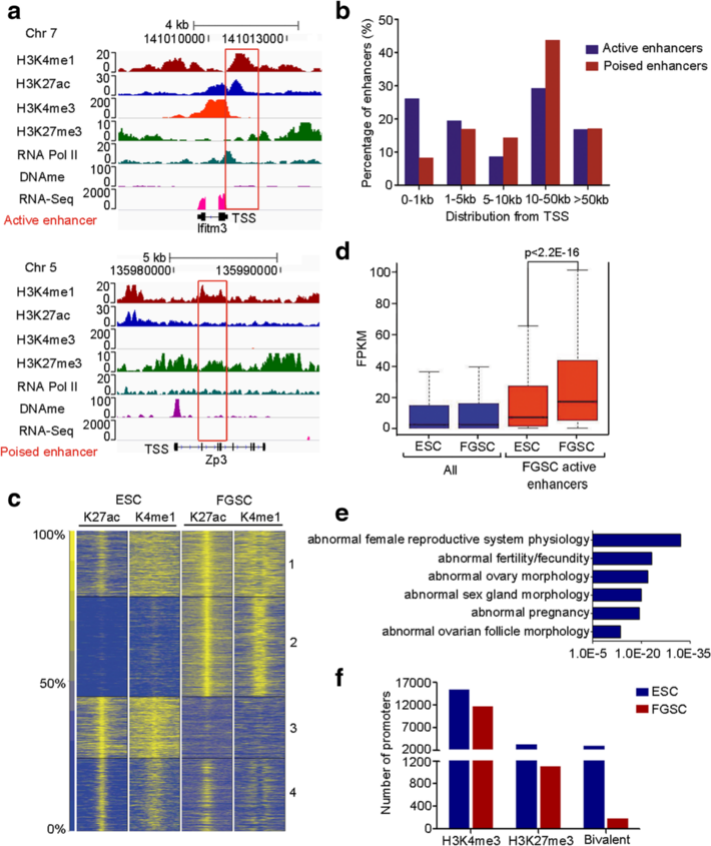
Epigenetic profiling identifies the enhancer regions in FGSCs. **a** Genome browser views of H3K4me1, H3K27ac, H3K4me3, H3K27me3, RNA Pol II, and DNA methylation (*DNAme*) enrichment profiles in FGSCs for an active (*Ifitm3*, *top red box*) or poised (*Zp3*, *bottom red box*) enhancer and the flanking regions. **b** Distribution of active and poised enhancers relative to their closest UCSC gene transcription start site (*TSS*). **c** K-means clustering of H3K4me1 and H3K27ac ChIP-Seq signals, the predictors of active enhancers, in ESCs and FGSCs. A window of 10 kb (−5 kb to +5 kb) around the peak center is shown. **d** Gene expression was measured as fragments per kilobase of exon per million fragments mapped (*FPKM*) and calculated for all mouse UCSC genes (*blue*) and for those closest (within 200 kb) to FGSC-specific active enhancers (*red*) (class 2 in **c**). Transcription levels in both cell types are presented as box plots (*p* values were calculated using paired Wilcoxon tests). **e** Enriched mouse phenotypes for nearest genes within 200 kb of FGSC enhancer signatures (*p* < 0.05). Loss of genes (e.g., *Npr2* and *Ptgs2*) with FGSC-specific enhancer signatures causes abnormal reproductive system physiology [57, 58]. **f** Number of bivalent promoters in ESCs and FGSCs

It has been recognized that enhancer regions are marked by H3K4me1 in a cell type-specific manner and involved in determining cellular identity [17, 24]. Moreover, these *cis*-regulatory elements could be further classified into “active” or “poised” enhancers based on the presence of H3K27ac [25]. Consistent with these observations, we found both types of enhancer sites in FGSCs (Fig. 2a). Examination of the genomic distribution of both types of enhancers relative to transcription start sites (TSSs) indicated that these enhancers exhibit a similar distribution pattern, with the majority located away from TSSs (Fig. 2b).

Both ESCs and FGSCs are capable of self-renewal in vitro, whereas they possess different developmental potential. To examine the underlying regulatory elements exclusively involved in FGSCs, we performed a K-means clustering analysis with active enhancer sites co-modified by H3K4me1 and H3K27ac and generated four major classes (Fig. 2c). Not surprisingly, genes associated with the FGSC-specific active enhancer regions (single nearest genes within 200 kb) exhibit higher transcriptional activity in FGSCs compared with ESCs (Fig. 2d). To understand how the lineage-specific enhancers contribute to FGSC identity, we performed gene ontology (GO) analysis with the Genomic Regions Enrichment of Annotations Tool (GREAT) [26]. We found the FGSC-specific enhancer peaks (class 2) are highly enriched for genes involved in reproduction-related phenotypes (Fig. 2e; Additional file 2: Table S2), including reproductive system physiology (e.g., *Notch2*, *Npr2*, and *Nr2f2*) and female fertility (e.g., *Ptgs2*, *Ptx3*, and *vrk1*) (Additional file 1: Figure S4a). Meanwhile, we found ESC-specific active enhancers (Fig. 2c, class 3) are mainly involved in embryogenesis and the active enhancers shared by ESCs and FGSCs are enriched for mitotic cell cycle-related genes (Additional file 1: Figure S4b).

### A bivalent domain chromatin signature is not widespread in FGSCs

Bivalent domain chromatin has been reported to be involved in developmental plasticity of ESCs [15]. Our previous study further revealed that the bivalent promoters are prevalent across the ESC genome [16]. In this study, we examined the presence of bivalent promoters in FGSCs. To our surprise, we observed that bivalent promoters in FGSCs are much less prevalent (Fig. 2f; Additional file 3: Table S3). It is less likely that this observation results from inefficient H3K27me3 ChIP-Seq as a considerable number of H3K27me3-marked regions are identified in FGSCs (Additional file 3: Table S3). A similar phenomenon was reported in multipotent neural crest cells [27]. These observations suggest that an “epigenetic code” other than the bivalent domain is responsible for the developmental plasticity of stem/progenitor cells.

### DNA methylation contributes to FGSC identity by suppressing the somatic program

**Fig. 3:**
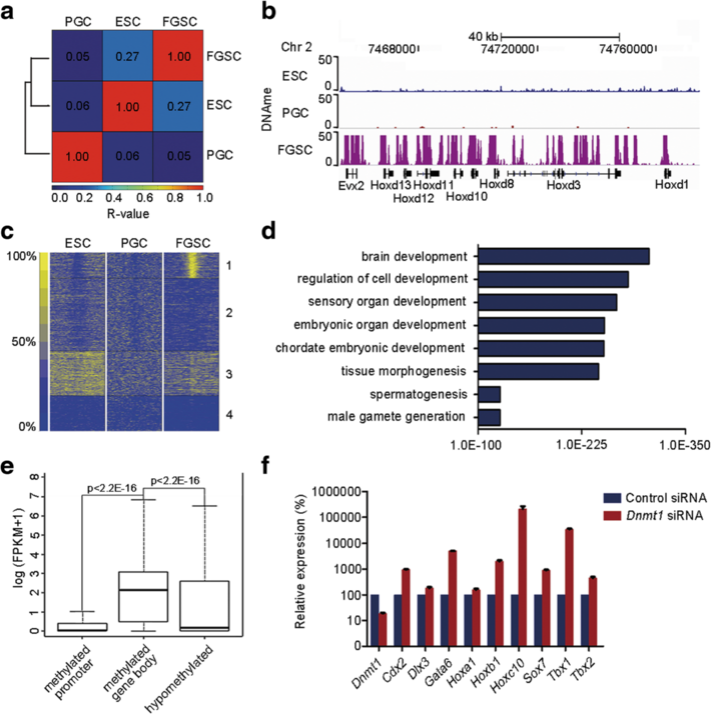
Genomic DNA methylation contributes to the identity of FGSCs. **a** Pairwise correlation comparison for genome-wide DNA methylation among distinct cell types. R values (Pearson correlation coefficient) were used to compare the significant correlation both within and between groups and is represented by a color scale (*red* is highly correlated). **b** An IGV snapshot of the DNA methylation state at the *Hoxd* gene family region. **c** K-means clustering of DNA methylation at promoter regions for ESCs, PGCs, and FGSCs. **d** Functional enrichment of FGSC-specific methylated regions by GREAT analysis (*p* < 0.05). **e** Expression levels of genes methylated at the promoter or gene body only or hypomethylated. **f** Quantitative RT-PCR analysis of development-related genes in *Dnmt1* knockdown FGSCs. Calculation of relative expression levels was based on comparison with the control. *Error bars* indicate standard deviations of three biological replicates

One of the major issues in germline stem cell biology is how unipotency is maintained. During the specification of germ cells, the Blimp1/Prmt5 complex plays an important role in the maintenance of unipotency through repressing targets by generation of repressive H2A/H4R3me2s and this complex translocates from the nucleus to cytoplasm in embryonic day (E)11.5 PGCs [28]. Moreover, we found that *Prdm1*, the gene encoding Blimp1, is not actively expressed in FGSCs. To understand how the unipotency of FGSCs is maintained, we examined the presence of DNA methylation, another epigenetic mark critically involved in gene silencing, across the FGSC genome. To this end, we generated a DNA methylation profile by MethylCap-Seq and compared our data with the data sets of the precursors of FGSCs generated by MeDIP-seq. We observed a remarkable difference in global DNA methylation patterns among ESCs, E11.5 PGCs, and FGSCs; the correlation between FGSCs and E11.5 PGCs is 0.05 and between FGSCs and ESCs is 0.27 (Fig. 3a, b).

To understand the significance of DNA methylation in FGSCs, we performed a clustering analysis with the data sets of ESCs, E11.5 PGCs, and FGSCs; the FGSC-specific methylated promoter regions (Fig. 3c, class 1) were used for GREAT analysis [26]. Notably, functional annotation revealed that the FGSC-specific methylated genes are mainly involved in somatic developmental processes (Fig. 3d), including Hox, Fox, and Tbx family transcription factors. Although viewed as a silencing epigenetic mark, growing evidence has revealed that the effect of DNA methylation on transcription is dependent on the genomic context [29]. We categorized the methylated regions of the FGSC genome into three groups and examined the relationship of DNA methylation and transcription activity. Similar to the previous study [30], we found that the transcription levels of genes with methylated promoters are lower than those of genes in the other two categories (Fig. 3e). Surprisingly, we observed that more than 90 % of genes with a methylated promoter are simultaneously methylated at the gene body (Additional file 4: Table S4), an epigenetic signature associated with active transcription [29]. To further explore the contribution of DNA methylation in FGSCs, we knocked down *Dnmt1* and found that several somatic development-related genes with DNA methylation and low occupancy of RNA Pol II at promoter regions were remarkably up-regulated (Fig. 3f). These results suggest that DNA methylation critically contributes to FGSC identity.

### Differential DNA methylation is involved in sexual identity maintenance of FGSCs

Germ cells are sexually bipotential in the early embryonic gonad and commit to either male or female development by E13.5 [31]. Although it is generally recognized that sex determination of germ cells is primarily determined by signaling molecules from the soma, increasing evidence has suggested the “sex” of the soma and germ cells must match each other for proper gametogenesis [32]. Nevertheless, it remains unclear how FGSCs intrinsically match the soma to maintain sexual identity; we thus asked whether DNA methylation is involved in this process.

**Fig. 4:**
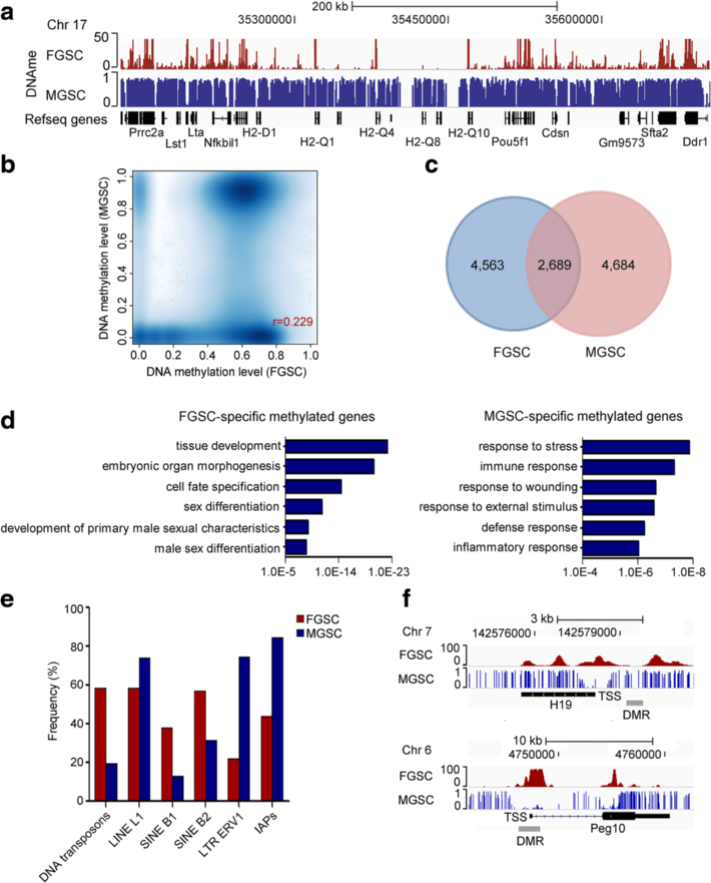
Comparison of DNA methylation state in FGSCs and MGSCs. **a** A snapshot of the IGV depicting DNA methylation in FGSCs and MGSCs. **b** Comparison of methylation at promoter regions (−2 kb to 500 bp) between FGSCs and MGSCs. We divided UCSC transcript promoters into 500-bp windows and showed that the absolute methylation signals from mean whole-genome bisulfite sequencing (MGSCs) and the mean MethylCap-seq normalized relative methylation levels (FGSCs) have a correlation of 0.229. **c** The number of genes methylated at TSS regions (−2 kb to 500 bp) in FGSCs and MGSCs. **d** Functional annotation of genes with FGSC-specific (*left*) or MGSC-specific methylation (*right*) (*p* < 0.05). **e** The DNA methylation frequency at transposable element loci. **f** DNA methylation status of imprinting genes (*H19* and *Peg10*) in FGSCs and MGSCs. *IAP* intracisternal A-type particle, *LINE* long interspersed nuclear element, *LTR* long terminal repeat, *SINE* short interspersed nuclear element

To address this issue, we compared the DNA methylation pattern in FGSCs with that in male germline stem cells (MGSCs) measured by bisulfite sequencing [33] (Fig. 4a). We analyzed the DNA methylation datasets with the method reported previously [34] and found the Pearson correlation to be 0.229 (Fig. 4b), suggesting a low correlation of DNA methylation between male and female GSCs. We particularly compared the methylated promoter regions. Among the methylated promoter regions of 11,936 genes, only 2689 (22.5 %) exhibit a similar DNA methylation level, whereas the majority exhibit a gender-specific methylation pattern (Fig. 4c; Additional file 5: Table S5). GO analysis of these female-specific methylated genes indicated they are involved in terms related to male sexual development (Fig. 4d). For example, *Bcl2l11*, *Nr0b1*, and *Sfrp2* play a critical role in development of male characteristics in mouse [35–37]. Here, we observed the promoters of these three genes are exclusively methylated in FGSCs and hypomethylated in MGSCs (Additional file 1: Figure S5). Transposable elements constitute 37 % of the mouse genome [38] and we investigated the DNA methylation of six categories of transposable elements that overlap with CpG islands. In contrast to the higher DNA methylation frequency of long interspersed nuclear element (LINE) L1, long terminal repeat ERV1, and intracisternal A-type particles (IAPs) in MGSCs, DNA transposons, SINE B1, and SINE B2 are more frequently methylated in FGSCs (Fig. 4e). We also investigated DNA methylation at imprinted loci. We observed the sex-specific DNA methylation pattern at differentially methylated regions (DMRs) of the imprinted loci examined (Fig. 4f; Additional file 1: Figure S6). Together, these results suggest DNA methylation is involved in sexual identity maintenance of FGSCs.

### *Prmt5* is implicated in FGSC biology

**Fig. 5:**
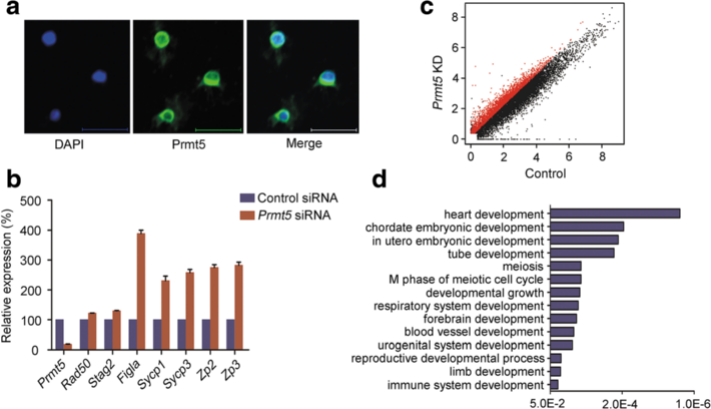
*Prmt5* is involved in FGSC biology. **a** Immunostaining of Prmt5 (*green*) in FGSCs. Nuclei were counterstained with DAPI (*blue*). Scale bar, 10 μm. **b** Quantitative RT-PCR analysis of meiosis-related genes in *Prmt5* knockdown FGSCs. Relative expression levels were normalized to the control. *Error bars* indicate standard deviations of three biological replicates. **c** Scatter plot of RNA-Seq reads in control (*x-*axis) and *Prmt5* knockdown (*y-axis*) cells. *Red dots* indicate genes that are up-regulated in *Prmt5* knockdown FGSCs. **d** GO analysis of up-regulated genes in *Prmt5* knockdown FGSCs (*p* < 0.05)

As mentioned above, Prmt5 forms a complex with the PGC determinant Blimp1 and is involved in the commitment of germ cell lineage, whereas the Blimp1/Prmt5 complex translocates from the nucleus to cytoplasm after E11.5 [28]. Our RNA-Seq data show *Prmt5* is actively expressed in FGSCs. Therefore, it remains intriguing to explore its function in FGSCs. Given the subcellular localization dynamics of Prmt5 during the early germline development, we first examined its localization and found that Prmt5 is primarily localized in the cytoplasm of FGSCs (Fig. 5a). We then performed a *Prmt5* knockdown assay (Additional file 1: Figure S7) and examined the biological consequences. We found that the meiosis-related genes (including *Figla*, *Sycp3*, and *Sycp1*) and oogenesis-related genes (including *Zp2* and *Zp3*) are up-regulated upon *Prmt5* knockdown (Fig. 5b), suggesting that *Prmt5* is involved in maintenance of the undifferentiated status of FGSCs.

To gain a global view of the effect of *Prmt5* on gene expression, we performed RNA-Seq analysis using *Prmt5* knockdown FGSCs and examined the *Prmt5*-responsive genes. Compared with the control, 2916 genes were found to be statistically up-regulated upon *Prmt5* knockdown (*p* < 0.05; Fig. 5c; Additional file 6: Table S6). Using DAVID [39], we performed GO analysis and found that, in addition to meiosis-related GO terms, some development-related biological processes (including heart development, embryonic development ending in birth or egg hatching, in utero embryonic development, developmental growth, and respiratory system development) are statistically enriched (*p* < 0.05) (Fig. 5d). These results suggest *Prmt5* is implicated in FGSC identity, possibly through suppressing both terminal differentiation and the somatic program.

## Discussion

Germline stem cells are critical for passing genetic information from generation to generation. In our previous work we generated mouse FGSCs [9] and several studies have reported the generation of FGSCs in other mammalian species [10–12, 40]. Although the existence of FGSCs has been debated and such a cell population possibly represents remnants of early PGCs or the gonocyte stage [41], in our study several lines of evidence indicate there is a big difference between FGSCs and PGCs. Firstly, several PGC determinants (including *Prdm1* and *Prdm14*) are not actively transcribed in FGSCs. Secondly, the transcriptome patterns of FGSCs and PGCs at various developmental stages differ to some extent (Additional file 1: Figure S8). Thirdly, although profound DNA demethylation occurs in late PGCs, we still observed obvious differences in DNA methylation patterns between E11.5 PGCs and FGSCs (Fig. 3a). These observations suggest it is less likely that FGSCs are remnants of PGCs.

Unipotency of FGSCs requires repression of the somatic program. Therefore, it is critical to understand how the somatic program is suppressed. Our previous work reported that the bivalent domain plays an important role in maintenance of ESC pluripotency, partially through transcriptional repression of the embryonic development program [16]. In this study, we found that such a bivalent domain chromatin signature is less prevalent throughout the FGSC genome. Instead, we observed that DNA methylation is actively involved in repression of the somatic program (Fig. 3d). Moreover, we found that DNA methylation of developmental genes is present only in FGSCs and not in MGSCs (Fig. 4d). Similar observations were reported in zebrafish germ cells [42], suggesting that such gender-specific DNA methylation patterns are probably conserved between teleost vertebrates and mammals. Among genes with a methylated promoter, more than 90 % are also methylated at the gene body region and most of these genes are involved in development (Additional file 4: Table S4). Given the lower presence of bivalent genes in FGSCs, we speculate that FGSCs utilize alternative epigenetic mechanisms, such as methylation at both promoters (the repressive transcription mark) and gene bodies (the active transcription mark), to maintain developmental plasticity. In addition to DNA methylation, we found *Prmt5* is also involved in such repression (Fig. 5d). These observations suggest that multiple layers of regulation restrict the somatic program in FGSCs.

The relatively simple characteristics of FGSC (self-renewal and unipotency) make it an ideal model for stem cell biology. Although the pluripotency genes *Nanog*, *Sox2*, *Esrrb*, and *Tcl1* [43] are repressed in both FGSCs and MGSCs [33], which suggests that GSCs may maintain unipotency by preventing the expression of the core pluripotency circuit, our recent work demonstrated that FGSCs could be converted into pluripotent stem cells [44]. Here, we found several mechanisms are shared in both types of stem cells. A previous report demonstrated that Prmt5 associates with Mep50 and methylates cytosolic histone H2A (H2AR3me2s) to inhibit differentiation of ESCs [45]. Similarly, our results indicate Prmt5 is involved in FGSC biology by repression of meiosis- and oogenesis-related genes (Fig. 5b, d). Although localized in cytoplasm, it remains to be elucidated whether Prmt5 forms a complex with Mep50 to exert the repression activity. In addition to Prmt5, Max was found to repress meiosis-related genes in ESCs and the expression of *Stra8* and *Sycp3* is significantly up-regulated in *Max* knockdown ESCs [46]. Consistent with this observation, we found the expression of some meiosis-related genes (including *Stra8*, *Sycp3*, and *Figla*) is significantly up-regulated when *Max* was knocked down in FGSCs (Additional file 1: Figure S9). These observations suggest FGSCs and ESCs share some mechanisms to inhibit differentiation. Given the conserved mechanisms in various types of stem cells, studies on FGSCs, despite their relative simplicity, may provide insights into the mechanisms involved in other types of stem cells with complicated properties.

## Conclusions

Our extensive epigenomic profiling analysis revealed that DNA methylation contributes to the unipotency of FGSCs primarily by suppressing somatic programs and is potentially involved in the maintenance of FGSC sexual identity. The genome-wide epigenetic signatures and the transcription regulators identified here provide an invaluable resource for understanding the fundamental features of mouse FGSCs.

## Methods

### Cell culture

FGSCs at passage number 32–35 were isolated as in our previous report [13] and cultured on gelatinized plates with minimum essential medium (MEM)-α containing 10 % fetal bovine serum, 1 mM non-essential amino acids, 2 mM L-glutamine, 1 mM sodium pyruvate, 0.1 mM β-mercaptoethanol (Sigma-Aldrich), 10 ng/ml mouse epidermal growth factor, 10 ng/ml mouse leukemia inhibitory factor, 40 ng/ml human glial cell line-derived neurotrophic factor, 1 ng/ml human basic fibroblast growth factor, and 15 μg/ml penicillin at 37 °C in a 5 % CO_2_ atmosphere as previously described [47].

### Immunofluorescence

Cells were fixed in 4 % paraformaldehyde for 15 min, washed twice in phosphate-buffered saline, and then incubated with 10 % goat serum at 37 °C for 15 min. Primary anti-Fragilis antibody (ab15592, Abcam, 1:200), anti-Mvh antibody (ab13840, Abcam, 1:100) or anti-Prmt5 (ab109451, Abcam, 1:50), and secondary antibody IgG (SA00003-2, Proteintech, 1:200) were used for immunostaining. Images were acquired using a Nikon A1Si confocal microscope or Leika DMI 3000 B inverted microscope.

### RT-PCR

Total RNA was extracted using Trizol reagent (Ambion) according to the manufacturer’s instructions. First-strand cDNA was transcribed using 4 μg total RNA and SuperScript™ III reverse transcriptase (Invitrogen) following the manufacturer’s protocol. We used a 20th of the RT reaction product for PCR amplification as described previously [9]. Primers for each gene are provided in Additional file 1: Table S7.

### ChIP-Seq

The preparation of ChIP and input DNA libraries were performed as previously described [27]. Briefly, cells were crosslinked with 1 % formaldehyde for 10 min at room temperature and quenched with 125 mM glycine. The fragmented chromatin fragments were pre-cleared and then immunoprecipitated with Protein A + G Magnetic beads coupled with anti-H3K4me1 (ab8895, Abcam), anti-H3K4me3 (ab8580, Abcam), anti-H3K27ac (ab4729, Abcam), anti-H3K27me3 (07–449, Millipore), and anti-RNA Pol II (ab5131, Abcam) antibodies. After reverse crosslinking, ChIP and input DNA fragments were end-repaired and A-tailed using the NEBNext End Repair/dA-Tailing Module (E7442, NEB) followed by adaptor ligation with the NEBNext Ultra Ligation Module (E7445, NEB). The DNA libraries were amplified for 15 cycles and subjected to deep sequencing with an Illumina HiSeq 2000.

### MethylCap-Seq

Methylated DNA was prepared with a MethylCap Kit (AF-100-0048, Diagenode) according to the manufacturer’s instructions. MethylCap and input DNA libraries were sequenced with an Illumina HiSeq 2000.

### Transcriptome analysis

Total RNA was extracted using Trizol (Ambion Life Technologies) according to the Ambion standard RNA isolation procedure and treated with TURBO DNase (AM2238, Ambion). Poly A^+^ RNA was purified using the Oligotex mRNA Mini Kit (70022, QIAGEN). Then the mRNA library was constructed with a NEBNext Ultra Directional RNA Library Prep Kit for Illumina (E7420S/L, NEB) and sequenced on an Illumina HiSeq 2000.

### RNA-mediated interference and quantitative RT-PCR analysis

Small interfering RNAs (siRNAs) targeting *Dnmt1*, *Prmt5*, and *Max* and control siRNA (Additional file 1: Table S8) were transfected using Lipofectamine 2000 (Invitrogen) according to the manufacturer’s instructions. Briefly, FGSCs were plated in growth medium without antibiotics one day before transfection. Transfection was performed at day 1 and day 3. For gene knockdown analysis, the cells were harvested 48 h after the second transfection and RNA was extracted using Trizol reagent according to the protocol provided by the manufacturer (Ambion Life Technologies). For reverse transcription, 5 μg of total RNA, 250 ng of random hexamer primers, and 200 U of SuperScript III (Invitrogen) were used in a 20-μl reaction volume. Quantitative PCR (qPCR) analysis was carried out with Maxima SYBR Green/ROX qPCR Master Mix (K0222, Thermo Scientific) on a StepOnePlus qPCR machine (Applied Biosystems).

### Data analysis

All histone modification ChIP-seq, DNA methylation, and control raw data reads were mapped using Bowtie (version 1.0.1) to the UCSC mm10 genome reference [48]. The normalized coverage was calculated by binning the unique tags in 50-bp bins and the number of reads in each bin was normalized using reads per kilobase per million reads (RPKM). We identified the enriched ChIP-Seq regions over background with the MACS version 1.4.2 (model-based analysis of ChIP-Seq) peak finding algorithm [49]. The parameters of MACS were set as default except --*nomodel* = T, --*shiftsize* = 75. In addition, the *p* value cutoff was set as 1.0E-3 for H3K27me3 calling. The DNA methylation data were processed with the MEDIPS method [50] with default parameters. To obtain the K-means results, we blurred the ChIP-Seq data with a rectangular mean kernel and applied a square root transformation to reduce the effect of noise and outliers. GO analysis was performed with GREAT [26] or DAVID [39].

For the purpose of comparison and clustering, other data were downloaded from the Gene Expression Omnibus (GEO) database. Mouse ESC ChIP-Seq data for H3K4me1, H3K4me3, and H3K27ac were obtained from Shen et al. [51] (GEO accession number GSE29218) and for H3K27me3 from Ng et al. [52] (accession number: GSE38164). The MeDIP-Seq and RNA-Seq data of mouse ESCs were obtained from Yu et al. [53] (GEO accession number GSE38596) and Surface et al. [19] (GEO accession number GSE53208), respectively. The E11.5 PGC MeDIP-Seq data were taken from Hackett et al. [54] (Sequence Read Archive accession number SRA060914). The adult male germline stem cell BiSeq data were obtained from Hammoud et al. [33] (GEO accession number GSE49624). The E9.5, E11.5, and E13.5 PGC RNA-Seq data were taken from Yamaguchi et al. [55, 56] (GEO accession number GSE41908).

For comparison of FGSC and MGSC DNA methylation datasets, we used the strategy in a previous report which allows comparison of methylation datasets generated by different approaches [34]. Briefly, we first divided the UCSC known gene promoter regions into 500-bp windows and calculated the methylation level in each window and then calculated the Pearson correlation coefficient between them. To examine the methylation levels in FGSCs, we evaluated them with the relative methylation score (rms), calculated with the R package MEDIPS; for the MGSC whole-genome bisulfate sequencing dataset, the absolute methylation signal (ams), calculated with the R package MethlKit, was used. To further analyze the difference, we divided the genome into 1-kb tiling windows and defined the highly methylated regions in each cell population. For FGSCs, the MEDIPS package was used and the regions that met the criteria (false discovery rate (FDR) adjusted *p* value <0.01 and fold change >2) were defined to be highly methylated. For MGSCs, the exact binomial test was used to test the significance of the ratio of C/(C + T); regions with a ratio significantly larger than 0.25 (FDR-adjusted *p* value <0.01) were regarded as methylated and those with a ratio significantly larger than 0.75 (FDR-adjusted *p* value <0.01) were regarded as highly methylated.

## Abbreviations

ChIP-Seq, chromatin immunoprecipitation sequencing; DMR, differentially methylated region; ESC, embryonic stem cell; FDR, false discovery rate; FGSC, female germline stem cell; GEO, Gene Expression Omnibus; GO, gene ontology; GREAT, Genomic Regions Enrichment of Annotations Tool; GSC, germline stem cell; MGSC, male germline stem cell; PGC, primordial germ cell; piRNA, piwi-interacting RNA; qPCR, quantitative polymerase chain reaction; RNA-Seq, RNA sequencing; RT-PCR, reverse transcription polymerase chain reaction; TSS, transcription start site

## Additional files

Additional file 1: Figures S1 to S9 and Tables S1, S7, and S8. (PDF 1366 kb) Additional file 2: Table S2. GO analysis of genes related to FGSC-specific active enhancers. (XLSX 10 kb) Additional file 3: Table S3. List of genes with bivalent promoters and histone modification regions (H3K4me3 or H3K27me3) in FGSCs. (XLSX 1417 kb) Additional file 4: Table S4. List of genes methylated at promoters or gene bodies. (XLSX 115 kb) Additional file 5: Table S5. List of genes methylated in FGSCs and/or MGSCs. (XLSX 180 kb) Additional file 6: Table S6. Genes up- or down-regulated in *Prmt5* kock-down FGSCs. (XLSX 733 kb)
