# Supplementary material for: Integrative epigenomic analysis reveals unique epigenetic signatures involved in unipotency of mouse female germline stem cells
Source: Genome Biol. 2016 Jul 27;17:162. doi: 10.1186/s13059-016-1023-z (PMC4963954; doi:10.1186/s13059-016-1023-z)
Supplement: Additional file 1: — Figures S1 to S9 and Tables S1, S7, and S8. (PDF 1366 kb) [file 13059_2016_1023_MOESM1_ESM.pdf]

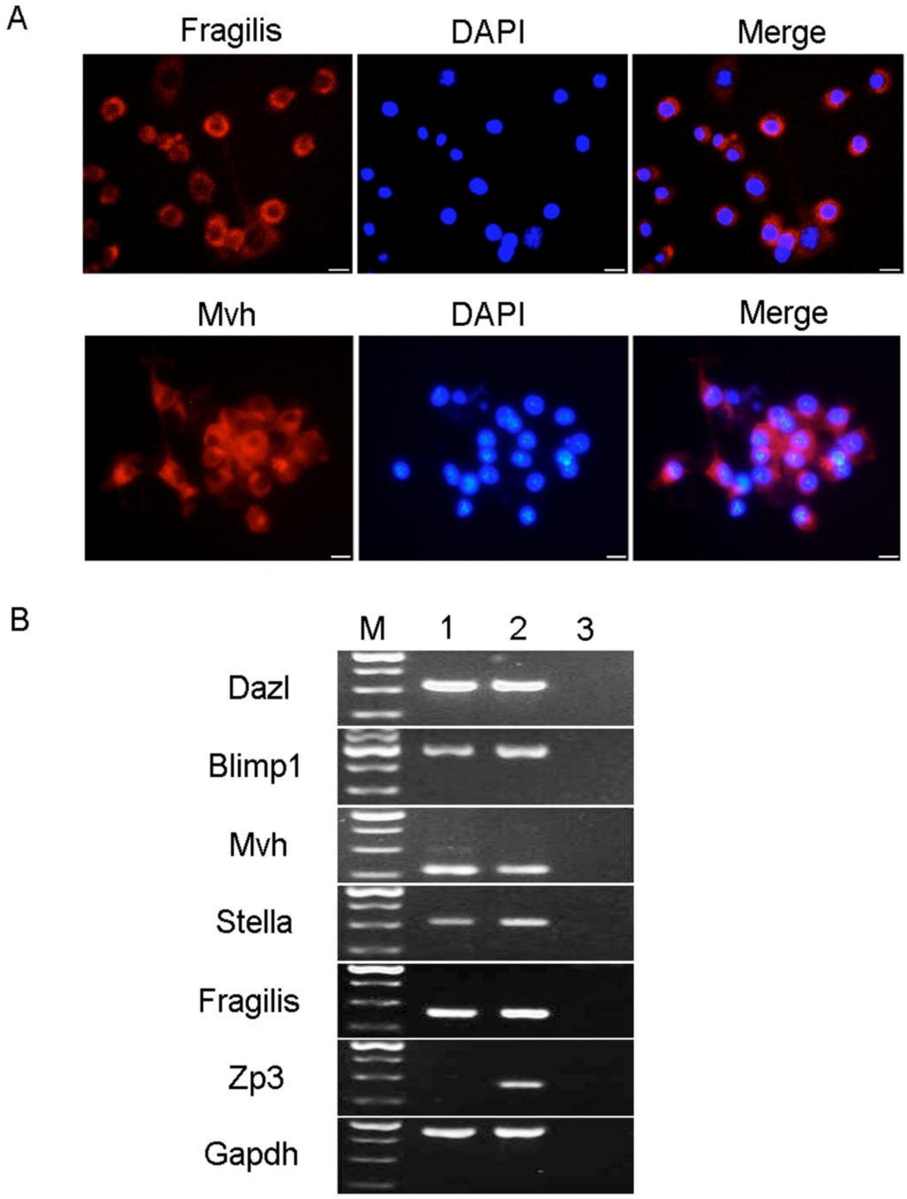

Figure S1. Characteristics of cultured FGSC.

(A) Immunostaining of Fragilis and Mvh (red) in FGSCs. Nuclei were counterstained with DAPI (blue). Scale bar, 10 μm. **(B)** RT-PCR analysis for the cultured FGSCs. M, 100bp DNA marker; lane 1, FGSC; lane 2, adult ovary; lane 3, mock-transcribed FGSC RNA samples. The size of the PCR product (in bp): *Dazl*, 328; *Blimp1*, 483; *Mvh*, 213; *Stella*, 308; *Fragilis*, 259; *Zp3*, 261; *Gapdh*, 458.

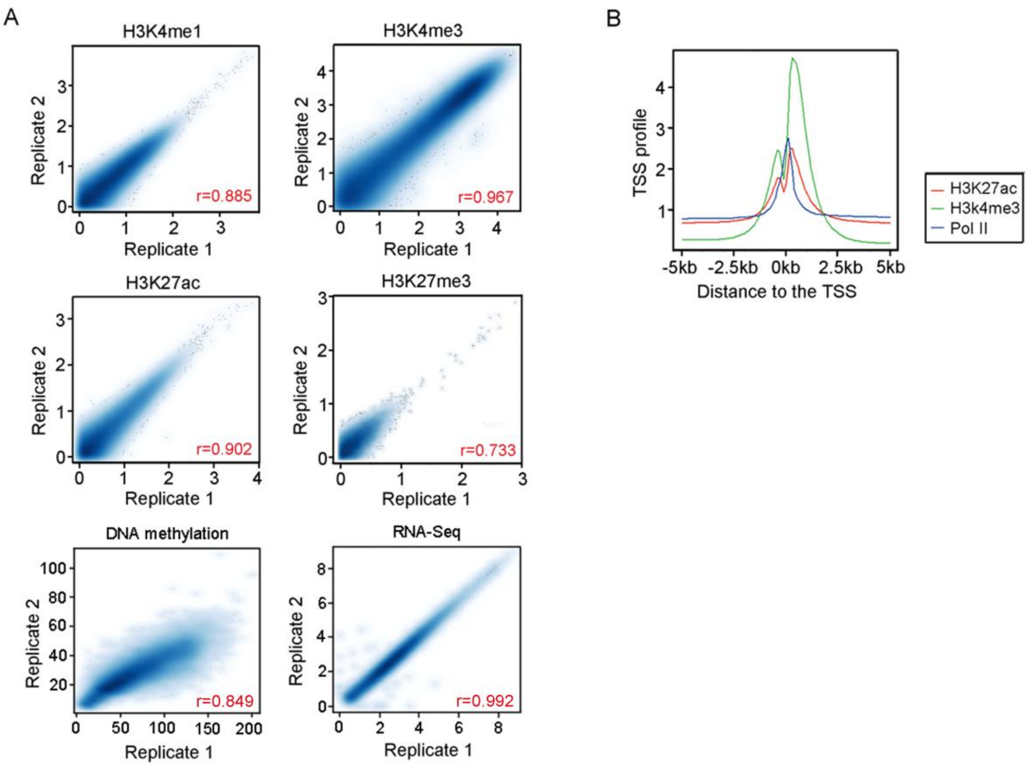

Figure S2. The repeatability and reliability of ChIP-seq data.

(A) Correlation analysis of replicate data, including ChIP-Seq (H3K4me1, H3K4me3, H3K27ac, H3K27me3), MethylCap-Seq and RNA-Seq. Correlation was computed using whole genome data. **(B)** Plot of relative FGSC H3K4me3, H3K27ac and RNA Pol II ChIP-Seq signal (normalized by mean genome-wide signal; within 5 kb of TSS) for all genes.

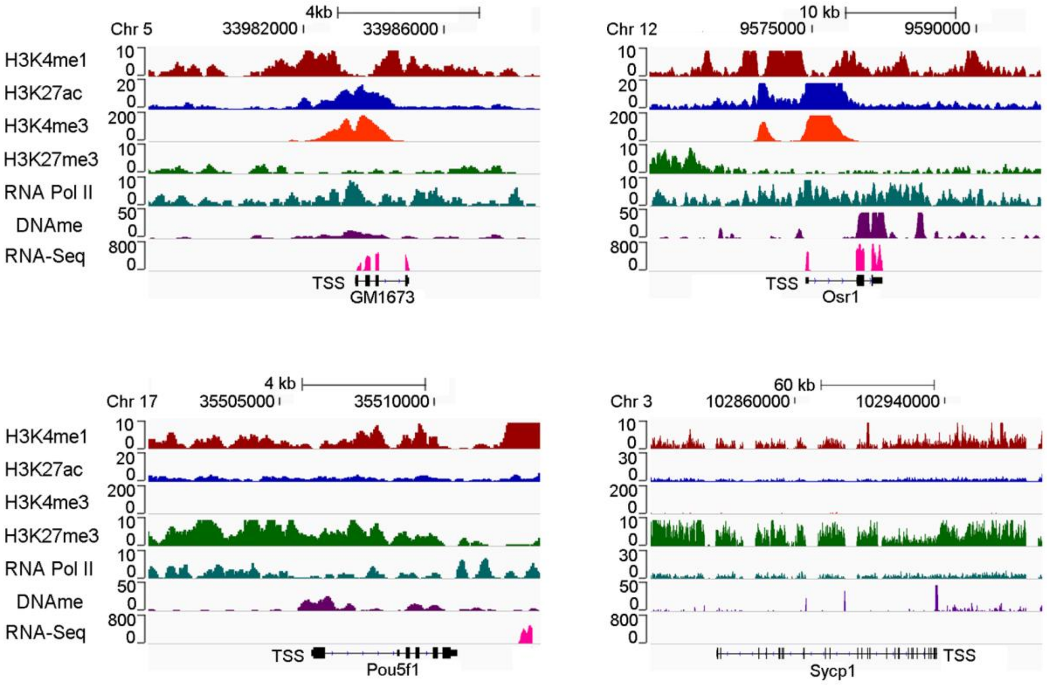

Figure S3. Epigenetic profiles of of important control genes in FGSC.

A snapshot of IGV browser depicting combined chromatin, RNA-Seq and DNA methylation profiles of FGSC genes (*GM1673*, *Osr1*, *Pou5f1*, *Sycp1*).

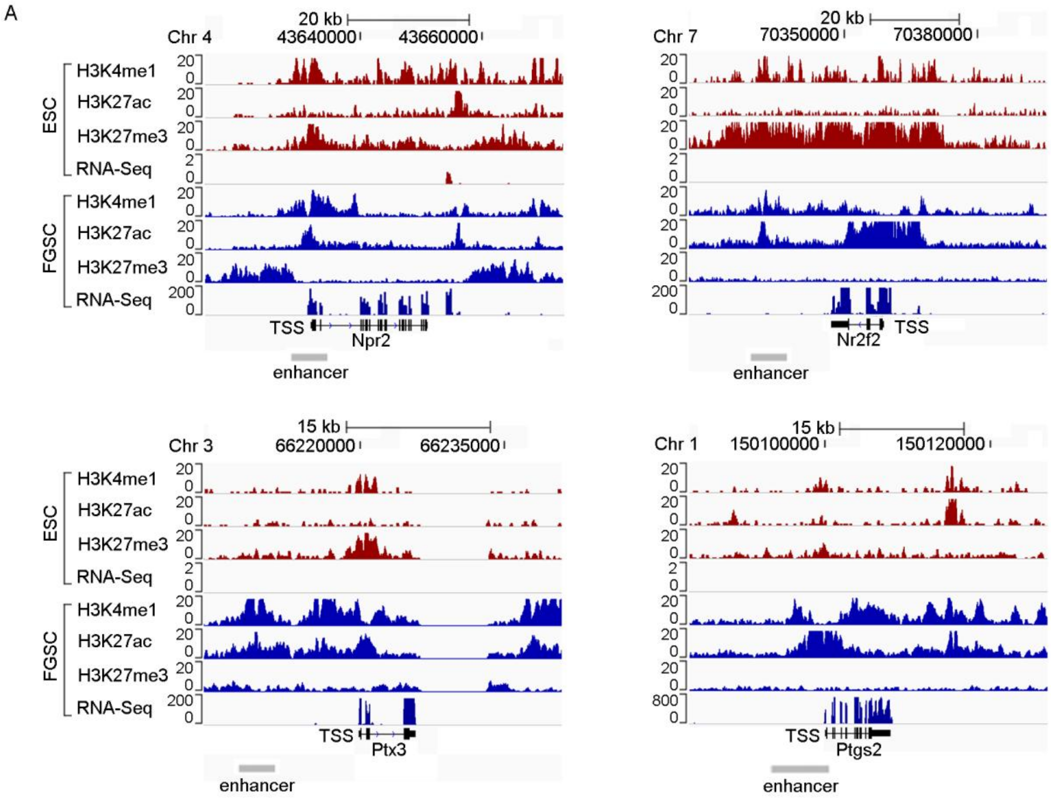

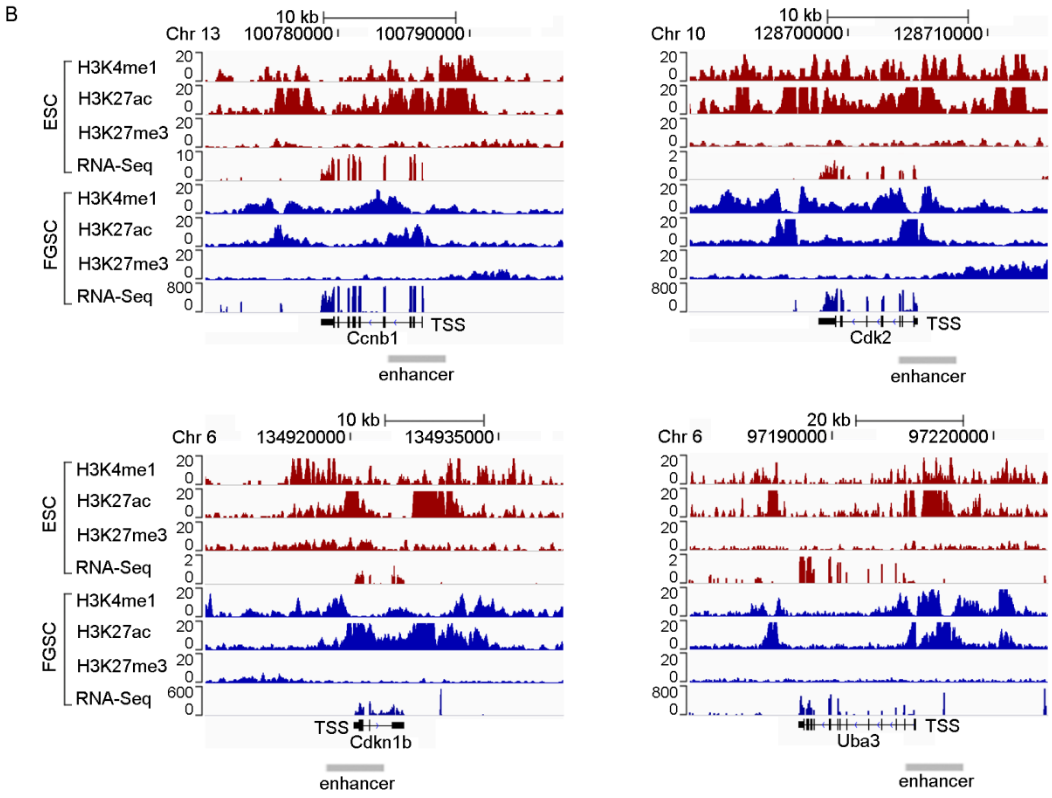

Figure S4. Epigenetic profiles of active enhancers.

(A) H3K4me1, H3K27ac, and H3K27me3 enrichment profiles in ESC and FGSC at representative FGSC-specific active enhancers. **(B)** H3K4me1, H3K27ac, and H3K27me3 enrichment profiles in ESC and FGSC at active enhancers shared between ESC and FGSC.

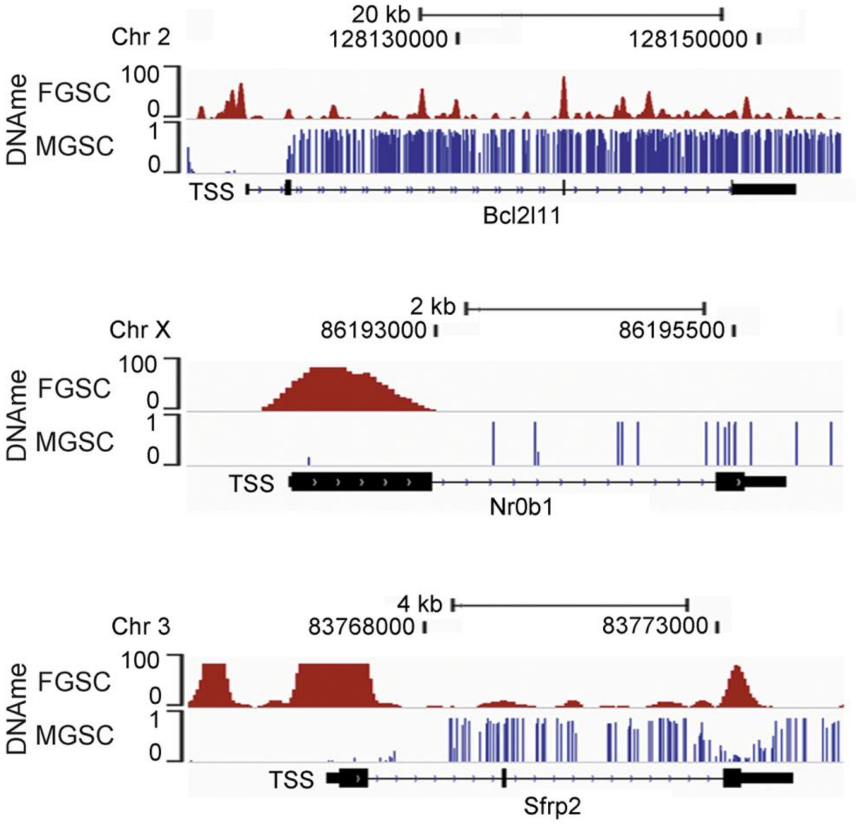

Figure S5. Browser snapshots depicting DNA methylation features of genes (*Bcl2l11*, *Nr0b1*, *Sfrp2*) in FGSC and MGSC.

Promoter regions of genes involved in male sexual development exhibit distinct methylation pattern in FGSCs compared with those in MGSCs.

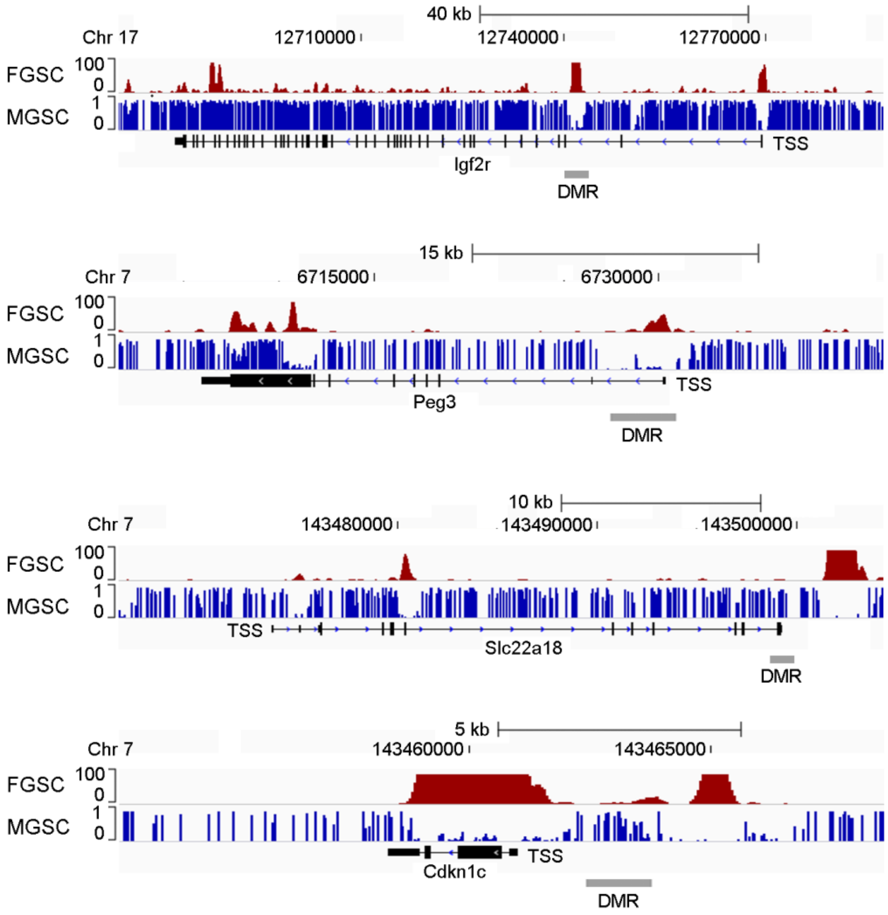

Figure S6. IGV browser view of DNA methylation shows the DMR of imprinting genes in FGSC and MGSC.

Maternally imprinting genes (*Igf2r*, *Peg3*) and paternally imprinting genes (*Slc22a18*,*Cdkn1c*).

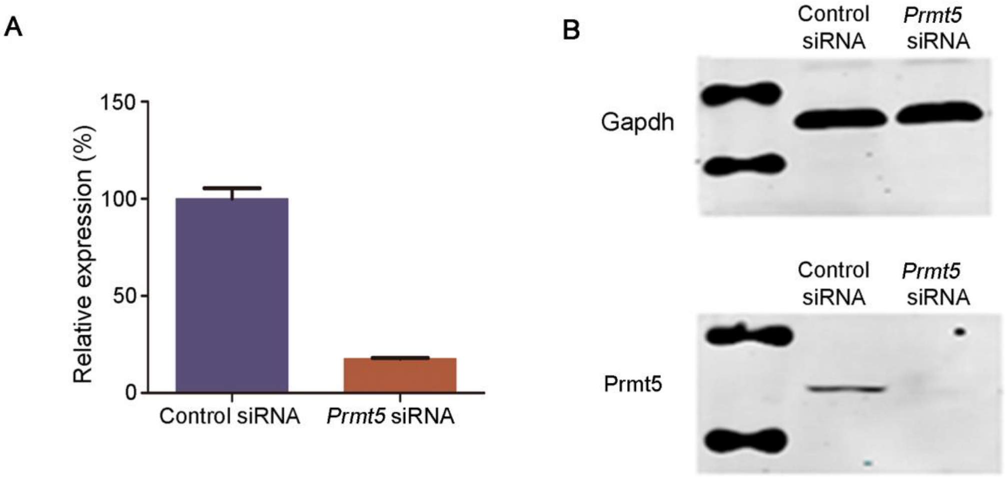

Figure S7. The detection of *Prmt5* knockdown efficiency.

(A) *Prmt5* knockdown efficiency in FGSC was assessed by qPCR. **(B)** Western blots depicting efficiency of *Prmt5* siRNA compared with control.

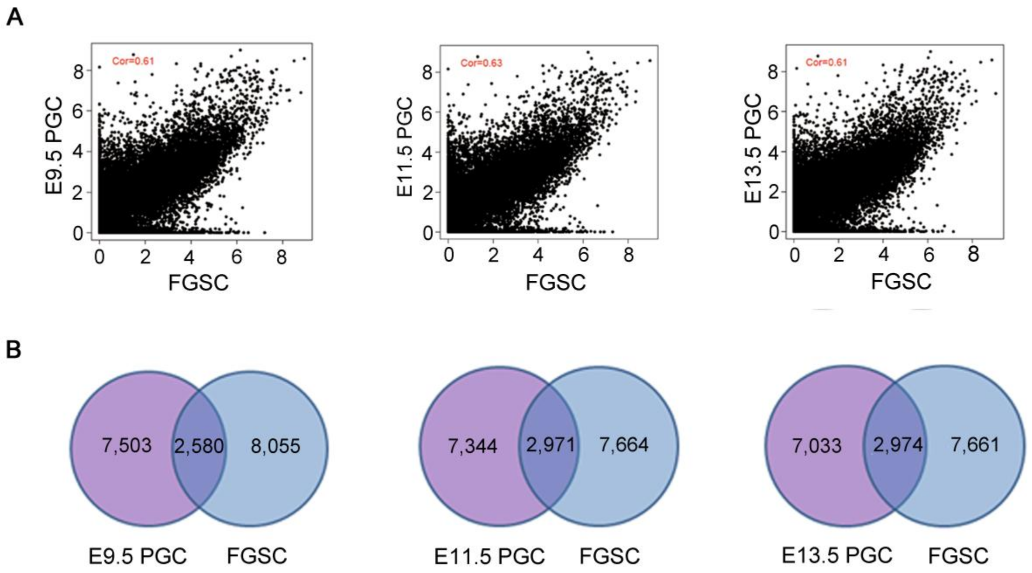

Figure S8. Correlations and differences of gene expression between PGC and FGSC.

(A) Scatter plots showing the correlation between the transcription levels of E9.5 PGC and FGSC (left), E11.5 PGC and FGSC (middle), and E13.5 PGC and FGSC (right). **(B)** Venn diagrams of the genes found to differentially expressed between E9.5 PGC and FGSC (left), E11.5 PGC and FGSC (middle), and E13.5 PGC and FGSC (right).

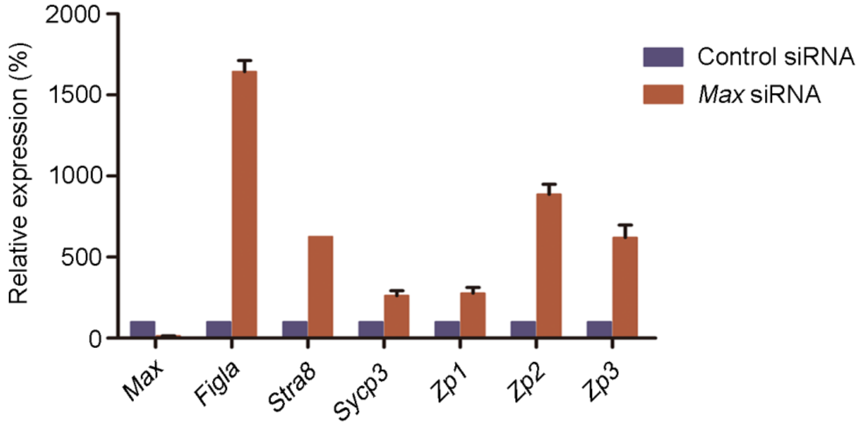

Figure S9. RT-qPCR analysis of *Max k*nockdown.

qPCR analysis of gene expression in *Max* knockdown cells harvested 5 d after knockdown. Levels of transcripts were normalized relative to control siRNA. Error bars indicate standard deviations of three biological replications.

Table S1. Detailed information on high throughput sequencing.

Sample	Details	Total reads	Unique reads
H3K27me3 ChIP-Seq	Biological replicate 1	33,924,419	23,698,079
	Biological replicate 2	21,734,504	15,852,340
H3K27ac ChIP-Seq	Biological replicate 1	20,921,145	15,713,879
	Biological replicate 2	35,793,074	27,913,051
H3K4me3 ChIP-Seq	Biological replicate 1	20,087,430	15,645,866
	Biological replicate 2	18,467,624	11,739,594
H3K4me1 ChIP-Seq	Biological replicate 1	28,078,381	22,423,370
	Biological replicate 2	20,690,576	16,442,339
RNA Pol II ChIP-Seq	Biological replicate 1	25,253,009	17,357,162
	Biological replicate 2	43,681,322	29,826,460
MethylCap-Seq	Biological replicate 1	45,183,069	17,242,232
	Biological replicate 2	36,698,682	14,999,661
ChIP-Seq input	Biological replicate 1	20,533,370	14,239,689
MethylCap-Seq input	Biological replicate 1	39,163,202	27,904,837
RNA-Seq	Biological replicate 1	59,848,781	50,966,714
	Biological replicate 2	66,783,558	57,773,948
Prmt5 KD RNA-Seq		25,845,978	3,788,822

Table S7. List of primers used in RT-PCR.

Genes	Primer Sequences (5'-3')
Dzal	F: GTGTGTCGAAGGGCTATGGAT
	R: ACAGGCAGCTGATATCCAGTG
Blimp-1	F: CGGAAAGCAACCCAAAGCAATAC
	R: CCTCGGAACCATAGGAAACATTC
Fragilis	F: TCCGTGAAGTCTAGGGATCG
	R: TGTTACACCTGCGTGTAGGG
Gadph	F: GTCCCGTAGACAAAATGGTGA
	R: TGCATTGCTGACAATCTTGAG
Mvh	F: GGAAACCAGCAGCAAGTGAT
	R: TGGAGTCCTCATCCTCTGG
Stella	F: GATGAAGAGGACGCTTTGGA
	R: TCCCGTTCAAACTCATTTCC
Zp3	F: GGGTGCAGATGACGAAAGAT
	R: TGGGAGCCGATTTCTCAGTA

Table S8. List of siRNA sequences used for RNAi.

siRNA	Sense (5'-3')	Antisense (5'-3')
Control	UUCUCCGAACGUGUCACGUdTdT	ACGUGACACGUUCGGAGAAdTdT
Dnmt1	GCUGGGAGAUGGCGUCAUAdTdT	UAUGACGCCAUCUCCCAGCdTdT
Max	CCACAUCAAAGACAGCUUUdTdT	AAAGCUGUCUUUGAUGUGGdTdT
Prmt5	GGAUGUGGUGGCAUAACUUdTdT	AAGUUAUGCCACCACAUCCdTdT
